# Supplementary material for: Mutations in PCYT2 disrupt etherlipid biosynthesis and cause a complex hereditary spastic paraplegia
Source: Brain. 2019 Oct 22;142(11):3382–97. doi: 10.1093/brain/awz291 (PMC6821184; doi:10.1093/brain/awz291)
Supplement: awz291_Supplementary_Data [file awz291_supplementary_data.zip › awz291-Suppl_data/Supplementary_Data4.zip!/Supplementary information 4 - plasma lipidomics.zip!/Plasma lipidomics/Control vs Patient2/Statistical plots and data/TG species/Volcano plot_alternative.pdf]

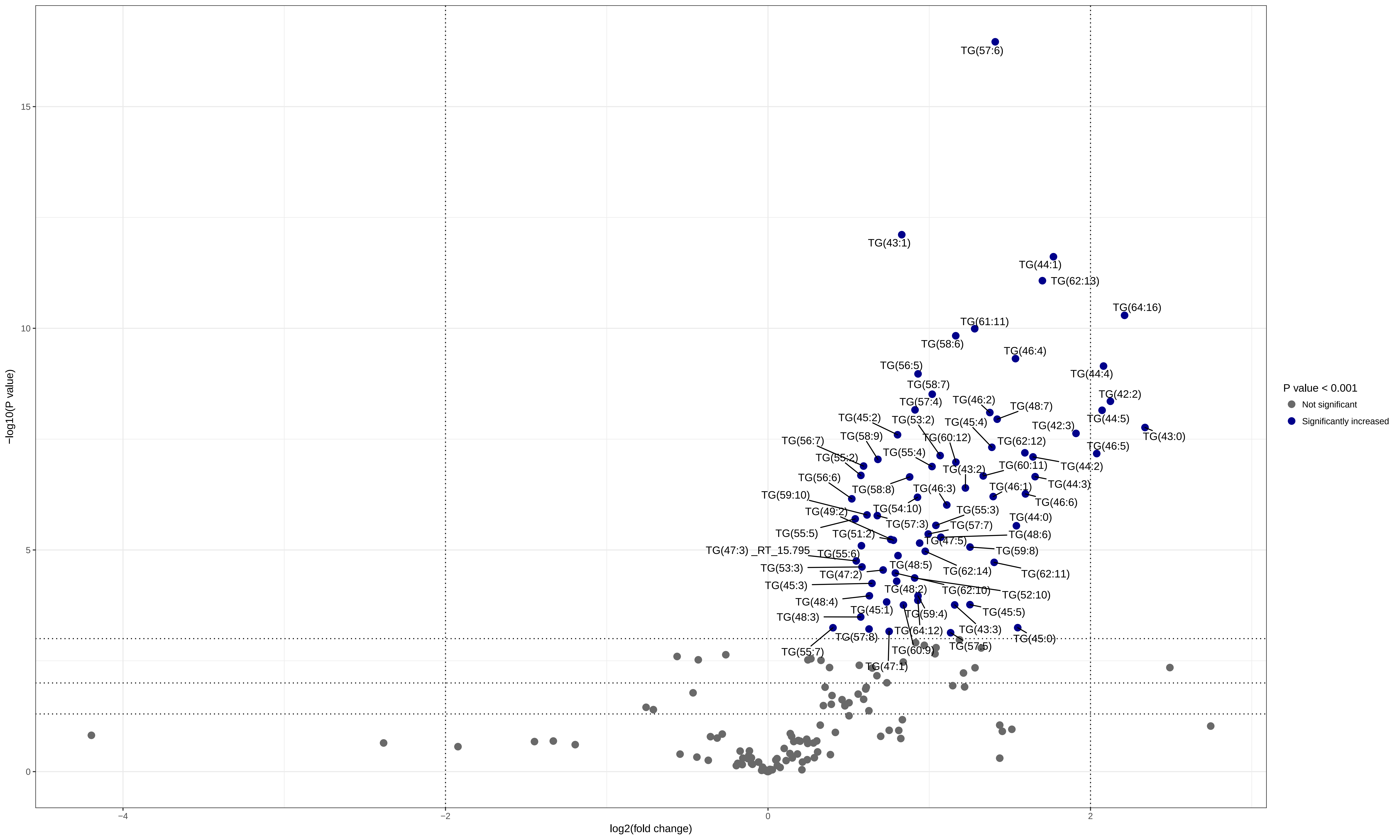

TG(57:6)
TG(43:1)
TG(44:1)
TG(62:13)
TG(64:16)
TG(61:11)
TG(58:6)
TG(46:4)
TG(56:5)
TG(44:4)
TG(58:7)
TG(42:2)
TG(57:4)
TG(46:2)
TG(48:7)
TG(44:5)
TG(45:2)
TG(53:2)
TG(45:4)
TG(42:3)
TG(43:0)
TG(58:9)
TG(60:12)
TG(56:7)
TG(62:12)
TG(46:5)
TG(55:4)
TG(55:2)
TG(44:2)
TG(43:2)
TG(60:11)
TG(56:6)
TG(44:3)
TG(58:8)
TG(46:3)
TG(46:1)
TG(59:10)
TG(46:6)
TG(54:10)
TG(49:2)
TG(55:3)
TG(44:0)
TG(57:3)
TG(57:7)
TG(55:5)
TG(51:2)
TG(48:6)
TG(47:5)
TG(47:3) _RT_15.795
TG(55:6)
TG(59:8)
TG(53:3)
TG(48:5)
TG(62:14)
TG(62:11)
TG(47:2)
TG(45:3)
TG(48:2)
TG(62:10)
TG(52:10)
TG(48:4)
TG(45:1)
TG(59:4)
TG(45:5)
TG(48:3)
TG(64:12)
TG(43:3)
TG(57:8)
TG(45:0)
TG(57:5)
TG(60:9)
TG(55:7)
TG(47:1)
15
10
5
0
−4
−2
0
2
−log10(P value)
log2(fold change)
P value < 0.001
Not significant
Significantly increased
